# Invasive Fungal Diseases in Kidney Transplant Recipients: Risk Factors for Mortality

**DOI:** 10.3390/jcm9061824

**Published:** 2020-06-11

**Authors:** Hyeri Seok, Kyungmin Huh, Sun Young Cho, Cheol-In Kang, Doo Ryeon Chung, Woo Seong Huh, Jae Berm Park, Kyong Ran Peck

**Affiliations:** 1 Division of Infectious Diseases, Department of Medicine, Samsung Medical Center, Sungkyunkwan University School of Medicine, Seoul 06351, Korea; hyeri.seok@gmail.com (H.S.); kyungmin.huh@samsung.com (K.H.); sunyoung81.jo@samsung.com (S.Y.C.); cheolin.kang@samsung.com (C.-I.K.); dr.chung@samsung.com (D.R.C.); 2 Division of Infectious Diseases, Department of Medicine, Korea University Ansan Hospital, Ansan 15355, Korea; 3 Division of Nephrology, Department of Medicine, Samsung Medical Center, Sungkyunkwan University School of Medicine, Seoul 06531, Korea; wooseong.huh@samsung.com; 4 Department of Surgery, Samsung Medical Center, Sungkyunkwan University School of Medicine, Seoul 06531, Korea; jaeberm.park@samsung.com

**Keywords:** invasive fungal disease, kidney transplantation, epidemiology, mortality, risk factor

## Abstract

Background: Invasive fungal disease (IFD) is common in solid organ transplant (SOT) recipients and contributes to high morbidity and mortality. Although kidney transplantation (KT) is a commonly performed SOT, data on the risk factors for IFD-related mortality are limited. Methods: A 1:2 retrospective case-control study was performed in an experienced single center in the Republic of Korea. We reviewed the electronic medical records of patients with IFD after KT between February 1995 and March 2015. Results: Of 1963 kidney transplant recipients, 48 (2.5%) were diagnosed with IFD. The median interval from KT to IFD diagnosis was 172 days. Invasive aspergillosis (IA) was the most common, followed by invasive candidiasis (IC). Diabetes mellitus (DM) (odds ratio (OR) 3.72, 95% confidence interval (CI) 1.34–10.31, *p* = 0.011) and acute rejection (OR 3.41, 95% CI 1.41–8.21, *p* = 0.006) were associated with IFD development. In the subgroup analyses, concomitant bacterial infection was associated with IC development (OR 20.10, 95% CI 3.60–112.08, *p* = 0.001), and delayed graft function was associated with IA occurrence (OR 10.60, 95% CI 1.05–106.84, *p* = 0.045). The 12-week mortality rate in all patients was 50.0%. Mortality rates were significantly higher in older patients (adjusted hazard ratio (aHR) 1.06, 95% CI 1.02–1.11, *p* = 0.004), or those with DM (aHR 2.61, 95% CI 1.02–6.68, *p* = 0.044), deceased donor transplantation (aHR 2.68, 95% CI 1.03–6.95, *p* = 0.043), lymphocyte-depleting antibody usage (aHR 0.26, 95% CI 0.08–0.80, *p* = 0.019), acute rejection (aHR 0.38, 95% CI 0.15–0.97, *p* = 0.044), and concomitant bacterial infection (aHR 8.76, 95% CI 1.62–47.51, *p* = 0.012). Conclusions: A total of 50% of IFD cases occurred six months or later after transplantation. The IFD-related mortality rate was high in kidney transplant recipients despite the low incidence. DM and acute rejection were associated with high mortality, as well as IFD development. As old age, deceased donor transplantation, lymphocyte-depleting antibody usage, and concomitant bacterial infection are risk factors for IFD-related mortality, efforts for its early diagnosis and appropriate treatment are required.

## 1. Introduction

Invasive fungal disease (IFD) is one of the critical opportunistic infections in solid organ transplant (SOT) recipients. IFD contributes to relatively high morbidity and mortality, compared to its low incidence, due to multiple causes, which include fungal virulence and delayed diagnosis in SOT patients [1,2,3,4]. The incidence of IFD among kidney transplant recipients is commonly known to be the lowest among SOT recipients, reported as 1–10% [2,5,6,7,8]. The period of the highest risk for the development of IFD is known to be the period of intense immunosuppression, from one to six months after SOT [9]. Recent studies reported that most IFD cases developed later than six months after kidney transplantation (KT) [5,8,10]. Although *Candida* spp. and *Aspergillus* spp. are common pathogens, the distribution of causative pathogens shows geographic variability [8]. Meanwhile, studies focused on IFD-related mortality in kidney transplantation (KT) are still limited [5,6,7,11,12]. We conducted an epidemiological and risk factor analysis of IFD incidence and IFD-related mortality in kidney transplant recipients.

## 2. Materials and Methods

### 2.1. Study Design and Population

We performed a retrospective case–control study at Samsung Medical Center in the Republic of Korea. Patients diagnosed with IFD after KT from February 1995 to March 2015 were selected as the case group. We matched two controls per patient; the control group comprised patients who underwent KT immediately before or after each case patient. Patients with superficial mucocutaneous candidiasis were not included in the study. The study protocol was approved by the institutional review board of the Samsung Medical Center (no. 2018-01-118).

### 2.2. Definition of IFD

IFD was defined according to the revised definitions of IFD of the European Organization for Research and Treatment of Cancer/Invasive Fungal Infections Cooperative Group and the National Institute of Allergy and Infectious Diseases Mycoses Study Group (EORTC/MSG) Consensus Group [12]. Mycological evidence included positive cultures from clinical specimens and indirect antigen tests. If specialists confirmed typical clinical signs of IFD based on the EORTC/MSG guidelines, possible IFD was diagnosed without mycological evidence.

### 2.3. Immunosuppression and Prophylactic Methods

Intravenous methylprednisolone was administered during surgery and tapered for 7 days. Anti-thymocyte globulin (ATG) was administered as induction therapy for 5 to 7 days when one of these conditions was satisfied: when re-KT; panel reactive antibody (PRA) >30%; donor-specific antibody-positive; or creatinine >2.0 mg/dL in deceased donor. Basiliximab was administered on the day of surgery and on postoperative day (POD) 4, starting in 2004. Kidney transplant recipients received a triple-immunosuppressive regimen, including calcineurin inhibitor, antimetabolites, and corticosteroids. Additional steroid pulse therapy or ATG was administered in cases of acute cellular rejection.

A single dose of trimethoprim/sulfamethoxazole was administered up to 6 months after KT. No systemic antifungal prophylaxis was applied. Ganciclovir was indicated for Cytomegalovirus (CMV)-seronegative recipients with a seropositive donor or ATG induction. Since 2000, preemptive ganciclovir therapy is performed for CMV antigenemia titers ≥50/4 × 10^5^ leukocytes with biweekly CMV screening within 3 months after KT and when a CMV infection is suspected [13].

### 2.4. Data Collection

The following patient data were reviewed: demographic information; comorbidities; information about KT, such as type of kidney donor, ABO incompatibility, human leukocyte antigen (HLA) matching, and renal replacement therapy (RRT) before KT; CMV serostatus; and immunosuppressive regimens. Acute rejection within 3 months before IFD and delayed graft function (DGF) after KT were checked. The diagnosis of acute rejection was confirmed by graft biopsy. DGF was defined as the use of dialysis within 7 days of transplant [14]. Graft failure was defined as the absence of kidney function requiring chronic dialysis and/or re-transplantation [15]. With respect to laboratory results, the most recent results were selected from the interval between the transplant and the infection, and the lowest values were selected for leukocytes when multiple tests had been performed in the month before IFD. Attributable mortality was defined as the total mortality in the study population minus the mortality in the population without IFD [16].

### 2.5. Statistical Analyses

Continuous data were described as mean ± standard deviation for normally distributed data and as median and interquartile range (IQR) for skewed data. All variables with any relevance to outcomes were evaluated by univariate logistic regression analyses, and those with statistical significance were included in the multivariate analyses. A Cox proportional hazard model was applied to identify associations between 12-week mortality and potential confounding factors. All analyses were 2-tailed, and *p* < 0.05 was considered statistically significant. All statistical analyses were performed using SPSS Statistics version 20.0 for Windows (IBM Corp., Armonk, NY, USA).

## 3. Results

### 3.1. Baseline Characteristics of Patients with IFD after KT

During the study, 48 (2.5%) out of 1963 kidney transplant recipients were diagnosed with IFD. The baseline characteristics are presented in Table 1. The median ages at KT and IFD diagnosis were 53 and 56 years, respectively. Of the patients with IFD, 24 were male. The most common cause of KT was diabetic nephropathy. Diabetes mellitus (DM) was the most common comorbidity and 23 patients had no additional comorbidities. The median dialysis time before KT was 1002 days (IQR, 444–2020 days), calculated from 43 patients.

The types and numbers of immunosuppressants are presented in Table 1. Lymphocyte-depleting antibodies were used in 36 patients and 8 patients received hemodialysis within seven days after transplantation due to DGF; 30 patients were treated for acute rejection before IFD. The median time from acute rejection to IFD was 42 days (IQR, 19–50 days). There were no donor CMV seropositive (D+)/recipient CMV seronegative (R-) cases. Concomitant bacterial infections were identified in 37 patients (77.1%).

### 3.2. Overall Characteristics of IFD in Kidney Transplant Recipients

The median time of onset of IFD after KT was 172 days (IQR, 38–1050 days). A total of 50% of IFD cases developed within six months (early IFD, *n* = 24) and 50% more than six months after KT (late IFD, *n* = 24). When comparing early and late cases of IFD, ATG use was significantly higher in early IFD (13 vs. 2, *p* = 0.001) and acute rejection was significantly more frequent in the late IFD group (7 vs. 16, *p* = 0.020). The median interval from fever or symptom onset to administration of the first antifungal agents was seven days (IQR, 4–13 days). No fever was detected in six patients. The median values of white blood cell count (WBC) and C-reactive protein (CRP) at the time of IFD diagnosis were 7360/μL and 8.2 mg/dL, respectively. The risk factors for the development of IFD are presented in Table 2. DM (odds ratio (OR) 3.72, 95% confidence interval (CI) 1.34–10.31, *p* = 0.011) and acute rejection (OR 3.41, 95% CI 1.41–8.21, *p* = 0.006) were associated with the development of overall IFD in the multivariate analysis. The median follow-up time for surviving patients was 2044 days (IQR, 780–3761 days).

The causative fungal species and the median time to onset of infections are presented in Table 3. Invasive aspergillosis (IA), the most common IFD, was diagnosed in 26 patients (54.2%). A total of seven samples of *Aspergillus fumigatus* and one each of *A. terreus*, *A. niger*, and *A. flavus* were isolated. A total of 17 patients (35.4%) were diagnosed with invasive candidiasis (IC). *Candida albicans* was the most common species, followed by *C. tropicalis*, *C. glabrata*, *and C. parapsilosis*. *Cryptococcus neoformans* was identified in two cryptococcal meningitis cases. *Trichosporon beigelii* was isolated in two urinary tract infections. *Scedosporium prolificans* was isolated from a subcutaneous abscess culture. Regarding the sites of infection, respiratory infections were the most common, occurring in 51.4% of cases, followed by intra-abdominal infections in 13.9%, and skin and soft tissue infections in 11.1%. *C. albicans* caused two cases of septic arthritis and *C. albicans* (*n* = 2) and *C. tropicalis* (*n* = 1) caused three cases of catheter-related bloodstream infections. Amphotericin B (*n* = 27), liposomal amphotericin B (*n* = 27), and fluconazole (*n* = 26) were commonly used antifungal agents.

The 12-week mortality rate in all IFD patients was 50.0% (*n* = 24), and 18 patients died because of IFD (attributable mortality, 37.5%). The risk factors affecting 12-week mortality rates are presented in Table 4. Age (adjusted hazard ratio (aHR) 1.06, 95% confidence interval (CI) 1.02–1.11, *p* = 0.004), DM (aHR 2.61, 95% CI 1.02–6.68, *p* = 0.044), deceased donor (aHR 2.68, 95% CI 1.03–6.95, *p* = 0.043), lymphocyte-depleting antibody usage (aHR 0.26, 95% CI 0.08–0.80, *p* = 0.019), acute rejection (aHR 0.38, 95% CI 0.15–0.97, *p* = 0.044), and concomitant bacterial infection (aHR 8.76, 95% CI 1.62–47.51, *p* = 0.012) were associated with increased 12-week mortality rates in the multivariate analyses. Neither early nor late IFD, nor the time from fever to the start of antifungal treatment were significantly associated with IFD-related mortality.

### 3.3. Characteristics of IC

The median time to diagnosis of IC after KT was 181 days (IQR, 38–3423 days). Of the 17 IC patients, 10 were diagnosed with intra-abdominal infections, followed by 3 with catheter-related bloodstream infections, 2 with infectious arthritis, and 2 with urinary tract infections. Fluconazole was the most commonly used empirical antifungal agent, employed in 11 patients, followed by echinocandin (*n* = 4) and amphotericin B (*n* = 2).

Age (OR 10.15, 95% CI 1.04–99.61, *p* = 0.047), DM (OR 5.16, 95% CI 1.34–19.78, *p* = 0.017), and concomitant bacterial infection (OR 15.17, 95% CI 3.64–63.12, *p = <* 0.001) were associated with the development of IC in the univariate analysis (Table 5). Concomitant bacterial infection was associated with the development of IC in the multivariate analysis (OR 20.10, 95% CI 3.60–112.08, *p* = 0.001).

The 12-week IC-related mortality rate was 52.9%, and five patients expired from IC (attributable mortality, 29.4%). One patient experienced graft failure 124 days after diagnosis of IC. The median follow-up time of surviving patients was 1777 days (IQR, 693–4580 days).

When 17 patients with IC were compared with 45 superficial candidiasis patients, preceding bacterial infections were significantly more common in the IC than in the superficial candidiasis group (76.5% vs. 20.0%, *p* < 0.001). The 12-week mortality rate was also higher among cases of IC than among those with superficial candidiasis (52.9% vs. 4.4%, *p* < 0.001).

### 3.4. Characteristics of IA

All 26 IA cases presented with invasive pulmonary aspergillosis (IPA). The characteristics of these 26 patients with IPA were published in our previous study and are briefly described here for comparison with the characteristics of IC [17]. The median time to diagnosis after KT was 161 days (IQR, 32–697 days). All patients were diagnosed as probable IPA. The median value of serum galactomannan was 0.71 (IQR, 0.45–4.05) in 23 patients. Age (OR 1.11, 95% CI 1.02–1.13, *p* = 0.006), DM (OR 4.03, 95% CI 1.37–11.91, *p* = 0.012), DGF (OR 12.14, 95% CI 1.34–110.29, *p* = 0.027), and acute rejection (OR 1.98, 95% CI 1.11–3.51, *p* = 0.020) were associated with development of IPA in the univariate analysis (Table 3). DGF was associated with IA development in the multivariate analysis (OR 10.60, 95% CI 1.05–106.84, *p* = 0.045).

Liposomal amphotericin B (*n* = 20, 76.9%) and voriconazole (*n* = 11, 46.2%) were the most commonly used antifungal agents. The 12-week mortality rate among IA cases was 57.7%. The median follow-up time of surviving patients was 1893 days (IQR, 590–2047 days).

### 3.5. Characteristics of Other Fungi

There were two cryptococcal meningitis cases. The cases developed 7 and 12 months after treatment for acute allograft rejection with steroid pulse therapy at POD 2616 and 1053, respectively. The first patient was also treated with ATG due to chronic rejection one month before admission for cryptococcal meningitis. *Cryptococcus neoformans* was identified from cerebrospinal fluid culture in both patients. The first patient expired within three days of antifungal treatment with amphotericin B. The second case is under outpatient follow-up without any complications of cryptococcal meningitis after successful treatment.

*Scedosporium prolificans* susceptible to itraconazole was isolated from the subcutaneous abscess culture of a 58-year-old male patient 99 days after KT. After debridement, itraconazole (100 mg twice a day) was administered for seven weeks until clinical signs improved.

## 4. Discussion

In this study, age, DM, decreased donor transplantation, lymphocyte-depleting antibody use, acute rejection, and concomitant bacterial infections were significant variables associated with the 12-week IFD-related mortality in kidney transplant recipients. While several studies have been published on the risk factors for development of IFD in kidney transplant recipients, studies on risk factors for mortality have been limited. Previously reported risk factors for mortality include organ damage, neutropenia, and steroid use [18]. These factors were not associated with mortality in our study. This difference may be attributed to the differences between the study populations. In the previously mentioned study, all SOT patients were included, where IC was the most common IFD (59%) and approximately 50% of IC cases developed within 100 days post-SOT. In contrast, only kidney transplant recipients were included, IC accounted for 35.4% of cases of IFD, and 50% of cases developed later than six months post-KT in this study. The risk factors for mortality, which may differ according to the organs transplanted, may be more informative when those are analyzed for each organ separately. As our study focused specifically on KT, it provides more significant data on the risk factors for mortality.

The incidence rate of IFD after KT was 2.5%, which is relatively low compared with those reported in previous studies (2.1–10.30%) [2,5,6,7]. The composition of causative organisms was different from prior studies. IA was the most common type of IFD in this study, when cases of superficial candidiasis were excluded from the total cases of candidiasis. Previous studies have not revealed the involvement of superficial candidiasis, but our result suggests that the rate of IC may be lower than expected. The previously known risk factors for IFD in SOT recipients were as follows: advanced age, DM, leukopenia, CMV infection, acute rejection, and intensive immunosuppression [1,11,19]. In this study, DM and acute rejection were associated with the development of IFD after KT, which is consistent with what was reported in previous studies. DM has been known to be associated with a high chance of fungal infection due to defects in cellular and humoral immunity as well as increased adherence of microorganisms to cells in patients with diabetes compared with that in patients with no diabetes [20]. Acute rejection is also associated with immune dysregulation including the inhibition of T helper type 1 response and enhancement of T helper type 2 response due to intensive immunosuppression [21]. We performed a subgroup analysis to define the risk factors according to fungal species, unlike previous studies, which did not differentiate between fungal infections by species. Concomitant bacterial infection and DGF were identified as risk factors for IC and IA occurrence, respectively. This suggests that the development of IA is more likely to be related to the immune status of the host compared with that of IC. In fact, 50% of IC cases developed six months after KT in this study, in contrast to within one month after transplantation in the previous study [2]. The risk factors for IC might be different with respect to time of occurrence after transplantation. Our data suggest that IC should be considered when bacterial infections are aggravated despite antibiotic treatment, regardless of the period after transplantation.

The crude 12-week mortality in this study was 50%, and the 12-week IC- and IA-related mortality rates were higher than 50%. Overall mortality was higher in this study than that reported in previous studies [7,11,18]. The risk factors associated with 12-week mortality in this study, i.e., age at IFD diagnosis, DM, deceased donor transplantation, lymphocyte-depleting antibody usage, acute rejection, and concomitant bacterial infections, had been previously known to be associated with IFD development, all related to the patients’ immune status. In particular, DM and acute rejection are notable in that they not only increased the chance of IFD but also that of mortality. The 12-week mortality rates in the IC subgroups were relatively higher than the 15–66% reported in previous studies [2,11]. This difference reflects the effort to exclude superficial candidiasis as mentioned above and suggests that the actual IC-related mortality rate may be higher. The IA-related mortality rate in this study was also higher than that reported in prior studies [18,22,23]. This suggests that screening for the early detection of IPA may be considered in kidney transplant recipients, especially in patients with recent changes in immune status. Although all IFD patients received the appropriate empirical antifungal treatment, the timing of antifungal treatment was not associated with an improvement in mortality. The association between antifungal treatment and IFD-related mortality still remains unclear and further studies are needed [24,25].

Our study has several limitations. First, the study included patients from within a long duration, resulting in heterogeneity in the study population. As the incidence rates of IFD were low in kidney transplant recipients, the study period had to be inevitably extended. To diminish the effects of the large study period and improvements in transplant medicine, we designed the control group to include kidney transplant recipients as close in time of KT as possible to each case group patient. Second, this is a single-center study. Although the 1963 kidney transplant recipients included in this study accounted for more than 10% of all KTs conducted in the Republic of Korea (17,525 cases from January 2000 to March 2015) [26], our results cannot be generalized. Third, the number of IFD cases was small despite the long study period. Although we tried to identify the overall characteristics of patients with IFD, the characterization of each fungal strain was limited due to small numbers. In particular, cryptococcosis, trichosporonosis, and scedosporiosis comprised only one or two cases; further research is needed on endemic and rare fungi.

In conclusion, IFDs after KT are not common, but are associated with high mortality rates. As approximately 50% of IFDs developed six months or more after KT, IFD, an uncommon disease, should not be neglected in kidney transplant recipients regardless of the duration after transplantation. For better outcomes, early vigilance and active efforts to diagnose IFD are important for kidney transplant recipients, especially in patients at risk of the development of and dying from IFD, even in a late period after transplantation.

## Figures and Tables

**Table 1:** Clinical characteristics of kidney transplant recipients with invasive fungal disease.

Characteristics	Case (*n* = 48)	Control (*n* = 96)	*p* Value
Age at Transplantation, Years; Median (Interquartile Range, IQR)	53 (37–58)	44 (34–54)	0.635
Age at IFD Diagnosis, Years; Median (IQR)	56 (39–60)		
Males	24 (50.0%)	61 (63.5%)	0.119
Pediatric Patients (<18 years of age)	2 (4.2%)	3 (3.1%)	1.000
Etiology of Chronic Kidney Disease			
Diabetes Mellitus	16 (33.3%)	12 (12.5%)	0.003
Adult Polycystic Kidney Disease	5 (10.4%)	3 (3.1%)	0.117
Hypertension	3 (6.3%)	14 (14.6%)	0.144
IgA Nephropathy	1 (2.1%)	16 (16.7%)	0.011
FSGS	0	4 (4.2%)	0.301
Other Glomerulonephritis	6 (12.5%)	9 (9.4%)	0.563
Others	3 (6.3%)	4 (3.1%)	0.686
Unknown	14 (29.2%)	34 (35.4%)	0.453
Diabetes Mellitus	17 (35.4%)	13 (13.5%)	<0.001
Dialysis-Dependence	43 (89.6%)	87 (90.6%)	0.563
Hemodialysis	34 (70.8%)	68 (70.8%)	1.000
Continuous Ambulatory Peritoneal Dialysis	9 (18.8%)	19 (19.8%)	0.882
ABO Incompatibility	11 (22.9%)	24 (25.0%)	0.784
Deceased Donor	32 (66.7%)	51 (53.1%)	0.121
Re-Transplantation	4 (8.3%)	2 (2.1%)	0.612
Induction Treatment			
Anti-Thymocyte Globulin	15 (31.3%)	44 (45.8%)	0.426
Basiliximab	13 (27.1%)	36 (37.5%)	0.214
Anti-Thymocyte Globulin + Rituximab	5 (10.4%)	5 (5.2%)	0.301
Alemtuzumab	1 (2.1%)	2 (2.1%)	1.000
Maintenance Immunosuppression			
Tacrolimus	32 (66.7%)	62 (64.6%)	0.804
Cyclosporine	7 (14.6%)	34 (35.4%)	0.009
Mycophenolate Mofetil	35 (72.9%)	87 (90.6%)	0.005
Azathioprine	1 (2.1%)	7 (7.3%)	0.269
Corticosteroid	42 (87.5%)	63 (65.6%)	0.005
CMV D^+^/R^-^	0	3 (3.1%)	0.551
Time post-Transplantation at Diagnosis of IFD			
Less than 1 Month	8 (16.7%)		
1 to 6 Months	16 (33.3%)		
6 to 12 Months	4 (8.3%)		
Longer than 12 Months	20 (41.7%)		
Delayed Graft Function ^†^	7 (14.6%)	6 (6.3%)	0.125
Acute Rejection Before IFD ^‡^	23 (47.9%)	28 (29.2)	0.027
Concomitant Bacterial Infection	37 (77.1%)		
Outcome			
Death within 12 Weeks	24 (50.0%)		
Graft Failure ^§^	7 (9.7%)	12 (12.5%)	0.088

^†^ Delayed graft function was defined as the use of dialysis within 7 days of transplantation. ^‡^ The diagnosis of acute rejection was confirmed by graft biopsy. ^§^ Graft failure was defined as the absence of kidney function requiring chronic dialysis and/or re-transplantation.

**Table 2:** Risk factors for the development of invasive fungal disease (IFD) in patients after kidney transplantation (KT).

Variables	OR (95% CI)	*p* Value	Adjusted OR (95% CI)	*p* Value
Age	1.04 (1.01–1.07)	0.005	1.03 (0.99–1.06)	0.101
Sex	0.57 (0.28–1.16)	0.121	0.61 (0.25–1.48)	0.274
Diabetes Mellitus	3.50 (1.49–8.21)	0.004	3.72 (1.34–10.31)	0.011
Dialysis-Dependence	0.72 (0.24–2.17)	0.564	0.48 (0.12–1.89)	0.292
ABO Incompatibility	0.89 (0.39–2.02)	0.784	0.81 (0.29–2.24)	0.678
Deceased Donor	1.77 (0.86–3.63)	0.123	2.10 (0.88–5.03)	0.095
Re-Transplantation	2.17 (0.46–10.27)	0.331	1.39 (0.27–7.08)	0.696
Lymphocyte-Depleting Antibody Usage *	1.05 (0.49–2.25)	0.898	0.46 (0.17–1.26)	0.132
Delayed Graft Function ^‡^	2.56 (0.81–8.10)	0.109	4.02 (0.74–21.98)	0.108
Acute Rejection ^§^	2.23 (1.09–4.58)	0.028	3.41 (1.41–8.21)	0.006

* Lymphocyte-depleting antibodies include anti-thymocyte globulin, basiliximab, and alemtuzumab, used according to each treatment indication. ^‡^ Delayed graft function was defined as the use of dialysis within 7 days of transplantation. ^§^ The diagnosis of acute rejection was confirmed by graft biopsy.

**Table 3:** Fungal pathogens causing invasive fungal disease.

Invasive Fungal Infection	No. (%) of Patients	Days from Transplantation
Invasive Candidiasis	17 (23.6%)	181 (38–3423)
*Candida Albicans*	5	
*Candida Tropicalis*	4	
*Candida Glabrata*	2	
*Candida Parapsilosis*	1	
Invasive Aspergillosis	26 (36.1%)	161 (32–697)
*Aspergillus Fumigatus*	7	
*Aspergillus Terreus*	1	
*Aspergillus Niger*	1	
*Aspergillus Flavus*	1	
Cryptococcosis	2 (2.8%)	1834 (1444–2225)
Trichosporonosis	2 (2.8%)	870 (445–1295)
Scedosporiosis	1 (1.4%)	99

**Table 4:** Risk factors for 12-week mortality in all IFD patients after KT.

Variables	Crude HR (95% CI)	*p* Value	Adjusted HR (95% CI)	*p* Value
Age at Diagnosis of IFD	1.03 (1.00–1.06)	0.038	1.06 (1.02–1.11)	0.004
Sex	0.75 (0.33-1.69)	0.489	0.68 (0.25–1.81)	0.435
Diabetes Mellitus	2.45 (1.10–5.46)	0.029	2.61 (1.02–6.68)	0.044
Dialysis-Dependence	4.39 (0.59–32.53)	0.148	2.13 (0.20–22.15)	0.528
ABO Incompatibility	1.49 (0.62–3.60)	0.373	0.65 (0.18–2.43)	0.525
Deceased Donor	1.88 (0.74–4.73)	0.182	2.68 (1.03–6.95)	0.043
Re-Transplantation	0.51 (0.09–2.79)	0.434	1.53 (0.21–10.98)	0.670
Lymphocyte-Depleting Antibody Usage *	1.02 (0.42–2.43)	0.969	3.84 (1.25–12.5)	0.019
Neutropenia ^†^	0.36 (0.05–2.67)	0.317	4.32 (0.13–147.01)	0.416
Delayed Graft Function ^‡^	1.27 (0.43–3.72)	0.664	0.66 (0.18–2.42)	0.533
Acute Rejection ^§^	0.72 (0.32–1.61)	0.419	2.63 (1.03–6.67)	0.044
Concomitant Bacterial Infection	4.66 (1.09–19.86)	0.038	8.76 (1.62–47.51)	0.012
Early or Late IFD	1.17 (0.53–2.62)	0.697	5.37 (0.57–50.32)	0.141
Timing Between Fever and Antifungal Treatment	0.97 (0.89–1.06)	0.513	1.00 (0.84–1.20)	0.970

* Lymphocyte-depleting antibodies include anti-thymocyte globulin, basiliximab, and alemtuzumab, used according to each treatment indication. ^†^ Neutropenia was defined as absolute neutrophil count (ANC) smaller than 500/uL. ^‡^ Delayed graft function was defined as the use of dialysis within 7 days of transplantation. ^§^ The diagnosis of acute rejection was confirmed by graft biopsy.

**Table 5:** Risk factors for invasive candidiasis and invasive aspergillosis (univariate analysis).

Variables	Invasive Candidiasis		Invasive Aspergillosis	
	OR (95% CI)	*p* Value	OR (95% CI)	*p* Value
Age	10.15 (1.04–99.61)	0.047	1.07 (1.02–1.13)	0.006
Male Sex	0.70 (0.22–2.26)	0.547	0.63 (0.24–1.62)	0.332
Diabetes Mellitus	5.16 (1.34–19.78)	0.017	4.03 (1.37–11.91)	0.012
Dialysis-Dependence		0.999	1.00 (0.27–3.69)	1.000
ABO Incompatibility	1.24 (0.26–5.96)	0.786	0.57 (0.19–1.66)	0.301
Deceased Donor	1.90 (0.55–6.57)	0.314	1.89 (0.71–5.00)	0.201
Re-Transplantation	4.40 (0.37–52.38)	0.241	1.62 (0.31–8.36)	0.567
Lymphocyte-Depleting Antibody Usage	0.88 (0.27–2.90)	0.839	1.00 (0.27–3.69)	1.000
Delayed Graft Function	1.00 (0.16–6.09)	1.000	12.14 (1.34–110.29)	0.027
Acute Rejection	1.13 (0.35–3.71)	1.131	1.98 (1.11–3.51)	0.020
Concomitant Bacterial Infection	16.25 (3.72–71.08)	<0.001		0.998
